# Durum Wheat Bread with a Potentially High Health Value through the Addition of Durum Wheat Thin Bran or Barley Flour

**DOI:** 10.3390/plants12020397

**Published:** 2023-01-14

**Authors:** Donatella Bianca Maria Ficco, Michele Canale, Virgilio Giannone, Maria Concetta Strano, Maria Allegra, Silvia Zingale, Alfio Spina

**Affiliations:** 1 Consiglio per la Ricerca in Agricoltura e l’Analisi dell’Economia Agraria—Centro di Ricerca Cerealicoltura e Colture Industriali, S.S. 673 km 25.200, 71122 Foggia, Italy; 2 Consiglio per la Ricerca in Agricoltura e l’Analisi dell’Economia Agraria—Centro di Ricerca Cerealicoltura e Colture Industriali, Corso Savoia 190, 95024 Acireale, Italy; 3 DSAAF—Dipartimento di Scienze Agrarie, Alimentari e Forestali, University of Palermo, Viale delle Scienze, Ed. 4, 90128 Palermo, Italy; 4 Consiglio per la Ricerca in Agricoltura e l’Analisi dell’Economia Agraria—Centro di Ricerca Olivicoltura, Frutticoltura e Agrumicoltura, Corso Savoia 190, 95024 Acireale, Italy; 5 Department Agriculture, Food and Environment (Di3A), University of Catania, Via S. Sofia 100, 95123 Catania, Italy

**Keywords:** durum wheat bread, barley flour, thin bran, physicochemical features, β-glucans, Mixograph, Farinograph, quality bread parameters, crumb color, crust color

## Abstract

The enrichment of semolina bread with prebiotic ingredients such as β-glucans may exert health-promoting effects. This work presents the results of a general recipe development aimed at improving the nutritional value of bakery products. In this study, increasing amounts (0%, 2%, 5%, 7%, and 10%) of thin bran or barley flour were added into re-milled durum wheat semolina to prepare breads. The technological quality of doughs and breads was investigated. In general, the Farinograph water absorption of flour and dough stability increased with increasing inclusion levels of barley flour or thin bran (up to 73.23% and 18.75 min, respectively), contrarily to the increase of dough development time only in barley inclusion (4.55 min). At the same time, the softening index decreased for almost all of these, except for 2% of thin bran or barley flour inclusion. At Mixograph, mixing time increased (up to 5.13 min) whilst the peak height decreased. The specific volume and hardness of loaf differently decreased for almost all thesis (ranges 12.6–24.0% and 39.4–45.5%, respectively). The other quality parameters remained unchanged compared with semolina bread. After baking, β-glucan levels increased differently at all the inclusion levels (2.35-fold, on average). The breadcrumb color was deep brown, while the crust became lighter in color. The breads contain β-glucans even at low percentages of barley/bran inclusions while maintaining their technological performance. In conclusion, the results show an interesting potential of barley flour or thin bran as ingredients in breadmaking to increase the β-glucans daily intake, but further investigations are needed to achieve improved quality features.

## 1. Introduction

Semolina is a typical cereal product of the Mediterranean region derived from the endosperm of the grain, presenting low levels of some valuable, healthy compounds, such as dietary fiber, vitamins, minerals, and antioxidants, which are abundant in bran [1]. Enriching wheat-based products, like bread, with health-promoting compounds, such as prebiotics, is becoming a common approach for the development of functional foods [2].

β-glucans are one of the soluble fractions of dietary fiber, non-starch polysaccharides that are the main components of the starchy endosperm and aleurone cells walls of cereals, with a role in the regulation of blood glucose and cholesterol levels [3]. β-glucans are most abundant in barley and oat (5–11% and 3–7%, respectively) and minimally in wheat grains (0.4–1.4%) [4]. Although barley is the richest cereal source, it has not been used in bakery products because of its appearance and taste factors, along with poor baking quality [5]. The main effect of β-glucans addition to bread is a reduced loaf volume as a consequence of water consumption of dough that increases proportionally to the partial substitution for wheat flour, influencing the rheological properties of dough [6,7]. Also, with the increase of β-glucan concentration, major viscosity was observed, as well as the reduction of shear rate and viscoelastic properties [8]. According to Ereifej et al. [9], the addition of barley flours up to 15% can improve the physicochemical properties of mixed bread by replacing wheat flour with barley flour. Alu’datt et al. [10] observed that 5–10% inclusion of barley flour in bread products did not change the texture and color of the products compared with the breads obtained with only wheat flour and was accepted by the consumers.

Moreover, β-glucan content and its properties like solubility, viscosity, and molecular weight are affected by different processing methods [11].

The higher dietary fiber content in bread than in flour has been reported previously [12]. Also, different forms of processing usually increase the extractability of dietary fiber: higher extractability is seen for β-glucan or arabinoxylan when studied separately, but not for total dietary fiber [13].

In general, the addition of bran to bread formulations—dependent on the type and level of bran incorporation—generally increases the dough water absorption, giving a heavier loaf and a reduced specific volume, with a darker color and reduced crumb softness [14,15]. In addition, some workers observed a mechanical effect of bran that physically disrupts gluten films, either during mixing or in the following steps of breadmaking and found in the fine grinding of bran a strategy to minimize this mechanical effect [14].

Barley and/or co-products of milling durum wheat, such as thin bran, are mainly used for animal feed and miscellaneous purposes, while only a small percentage is destined for human consumption [16,17]. The primary reason for this disparity in the use of these cereals is due to the remarkable visco-elastic nature of wheat dough; so, the desired dough properties were achieved using wheat in conjunction with barley or other cereals [17,18]. Therefore, a comparative investigation of different cereal flours with a special focus on composition and flour performance properties is needed. Similarly, some authors have also studied the impact of adding barley and other cereal flour on wheat bread quality [19,20].

This study has been designed to focus on the dough’s rheological and bread-making quality when supplemented with increasing concentrations (0%, 2%, 5%, 7%, and 10%) of barley or thin bran flour to improve the nutritional quality of bread.

## 2. Results and Discussion

### 2.1. β-Glucans and Chemical Characterization of Semolina and Flour Blends

The incorporation of β-glucan in foods such as bread and pasta, seen as highly healthy by the consumer [21], through the use of barley flour [22] in progressive replacement of wheat flour [23] has been widely studied, as regards the ability to interfere on the physicochemical and rheological properties of doughs.

The analyses conducted in our study showed high β-glucan contents in 100% barley flour, in line with what was found by other authors [24,25,26], equal to 10.61% (Table 1). As regards the content of β-glucan in semolina (0.31%), it has clearly lower values than 100% barley flour, tending to increase in content in relation to the presence of the bran fraction present as observed by other authors [27], confirming the values we reported for 100% thin bran (1.19%).

Naturally, the addition of barley flour in the various supplements resulted in an increase in the content, measured in the 10% supplemented semolina (1.58%), in β-glucan equal to five times compared to the 100% control re-milled semolina.

This differs from what was found in thin bran, which did not contribute significant quantities of β-glucan in the various additions. These data agreed with Basman et al. [28], who, studying the effect of barley flour and wheat bran supplementation on the composition of Turkish flatbread, observed only a significant increase in β-glucan values with an increasing percentage of barley flour.

Moisture, protein content, and ash of semolina fulfilled the legal requirements (Italian Presidential Decree n. 187/2001) and were in the range observed by [15,29]. Moisture did not show differences among barley flour or thin bran inclusions, whilst resulting lowest in barley flour and the highest in thin bran whole flours. Protein content decreased significantly in whole barley flour and different barley blends at increasing percentages of inclusion compared with semolina, accordingly to Mohebbi et al. [30]; on the contrary, in thin bran, protein content was higher than semolina, at increasing levels of inclusion. Otherwise, in wheat flour complemented with different amounts of oat flour (5%, 15%, and 25%), the protein content was similar with up to 15% oat inclusion [31]. These data confirmed that a relatively greater proportion of proteins among cereals was found in wheat flours (10.18–11.25%) compared with barley flours (7.09–9.04%) [29,32].

Similar to durum wheat thin bran, barley flour is also a source of ash. The aleurone cells, together with the testa and germs, contain essential minerals required for embryo growth [33]. The highest ash values were observed for whole thin bran and whole barley flours. The mean ash content increased with an increase in barley or thin bran inclusions, remaining below the legal limits of Italian law for semolina (maximum ash content of 0.9%). The data observed were in line with those reported by Mehfooz et al. [34] for wheat flour/barley blends.

**Figure 1:**
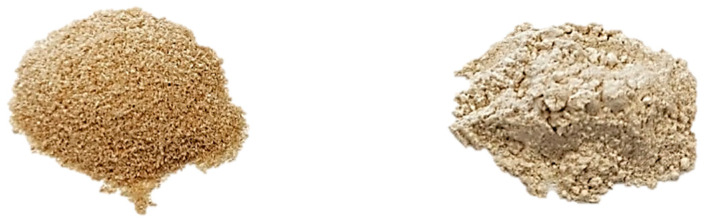
Thin bran (**left**) and barley flour (**right**).

Regarding the color indices (Table 1, Figure 1), significant differences in the brown index were observed for whole thin bran flour and 10% thin bran inclusion compared with semolina, whilst the red index was less negative in whole barley flour and highly positive in whole thin bran flour; finally, significant differences in the yellow index were observed only between whole barley and whole thin bran flours. These data were in accordance with Basman et al. [28].

Table 2 shows a two-factor ANOVA (analysis of variance) of the physicochemical features of the thin bran and barley flour.

Almost all variables, except β-glucan content, which is about six times higher, show higher values in thin bran.

Table 3 shows a two-factor ANOVA (analysis of variance) of the physicochemical features to the different percentages of integration of two flours.

β-glucan increases with the increasing percentage of supplementation until it reaches 5.9% in 100% supplementation.

The technological properties of dough from barley- or thin bran-semolina flour blends compared with semolina were determined by Mixograph and Farinograph tests (Table 4). Mixing time (namely, the time in minutes required for optimum dough development) increased in barley with the increasing percentage of inclusion. The results were in line with those reported by Tömösközi et al. [35], who observed an increase in the mixing time and a decrease in protein content because of the addition of non-wheat protein fractions. Instead, in thin bran inclusions, higher mixing time values were observed for 2% and 10% and lower values for 5% and 7% as compared with semolina. Different mixer inclusions probably influence the mixing procedures and dough properties differently. Peak dough height (i.e., a measure of dough strength) was lower in barley and thin bran formulations compared with semolina, with different behaviors except for 2% barley and 10% thin bran, which were similar to semolina. In particular, barley at the major percentage of inclusion corresponded lower peak dough height compared with semolina; the contrary was observed for thin bran. The high height values can probably be attributed to the influence of flour protein content on flour water absorption, so with increasing protein content, doughs became stiffer, resulting in increasing Mixograph peak height values. The opposite was for dough at low protein content. A positive relationship between protein content and Mixograph peak height (*r* = 0.60 **) was observed, and it was consistent with previous studies [36]. Mixograph peak height may provide an important quality criterion in assessing flour quality performance, especially in early-generation selection.

Regarding the Farinograph parameters, water absorption (i.e., the percentage of water required to reach a dough consistency of 500 Brabender Units) progressively increased as the amount of barley and thin bran integration added increased, and barley inclusion caused a greater increase (from 63.05% to 73.23% in barley and from 63.68% to 65.32% in thin bran, respectively). The increase of hydration capacity was different between flour inclusions, which could be explained by the intake and type of fibers that barley or bran brought into the dough, also determining higher yield in breads [37]. This result has been related to the high-water absorbing capacity of the dietary fiber and its ability to compete for water with other components in the dough system, interfering with the formation of a strong gluten network and dough stability [26]. Similar to Kaur et al. [36], a strong relationship (*r* = 0.80 **) between Mixograph peak height and Farinograph absorption was evidenced, which could be due to the impact of proteins as well as other factors such as starch damage and gluten strength which can strongly influence Farinograph absorption.

Dough development time (i.e., the time needed from the first addition of water to reach the maximum consistency) did not have a clear-cut trend with the increase of barley or bran flour supplementation. In addition, bran integrations did not show statistically significant differences compared with semolina (*p* < 0.01), except for 2% bran inclusion, showing the lowest development time. The results were in line with Popa et al.’s findings [37]. The development time of different kinds of flours could be strongly influenced by protein content and quality, as evidenced by other authors [30,38].

Dough stability (i.e., the difference between the time needed to reach the dough consistency of 500 Brabender Units and the time when it leaves the 500 B.U. line) was maximum at 5% added of barley flour (10.08 min), then tended to decrease at larger additions of barley flour. Instead, it was high and constant from 5–10% bran inclusion (18.75 min, on average), reaching equal value to the control at 2% level (3.78 min). The different effects in dough stability among samples could depend on the development of stiffer dough with the increase in barley (reaching a plateau at 5% inclusion) or bran (at 5–10% inclusions) due to more amount of water being absorbed by fiber [34]. Since stability is correlated with dough tolerance to kneading, fermentation, and even with the volume of finished products, the evolution of this parameter can be used to determine the optimal amount of flour inclusion.

Finally, the softening degree (i.e., the loss of dough consistency after 12 min) significantly decreased in all samples compared with semolina, except for 2% levels in barley and thin bran. The reduction of softening index could be due to a dilution of gluten by bran leading to dough deterioration, breaking of the starch–gluten network structure determining a decrease of consistency, with the release of water from the system [39]. On the other hand, the discrepancies related to the influence of fiber on the dough may arise from differences in the molecular size, solubility, and concentration range of the polysaccharides, as well as the flour types used for supplementation [40].

Table 5 shows a two-factor ANOVA (analysis of variance) of the main technological parameters of the thin bran and barley flour. All variables, except dough stability, show higher values in barley flour.

Table 6 shows a two-factor ANOVA (analysis of variance) referring to the different integration percentages of two flours: thin bran and barley flour. As far as the Mixograph is concerned, as the percentage of integration increases, the mixing time increases, and the peak dough height decreases.

The Farinograph parameters increase as the integration percentage increases, except for development time and dough stability at a 10% inclusion level.

### 2.2. The Quality Parameters of Breads Using Different Formulations

**Figure 2:**
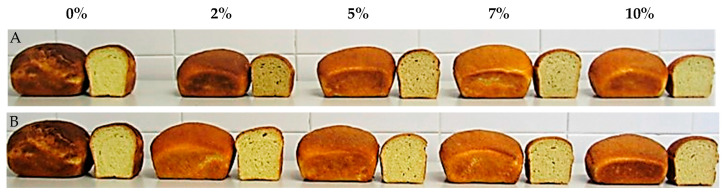
Experimental groups of bread loaves with (**A**) 0%: semolina sample (ctrl), 2%: 2% barley flour powder addition, 5%: 5% barley flour powder addition, 7%: 7% barley flour powder addition, 10%: 10% barley flour powder addition or with (**B**) 0%: semolina sample (ctrl), 2%: 2% thin bran flour powder addition, 5%: 5% thin bran flour powder addition, 7%: 7% thin bran flour powder addition, 10%: 10% thin bran flour powder addition.

Significant differences (*p* < 0.01) in specific volume were shown among the bread samples, with slight changes across the type and level of supplementation (Table 7, Figure 2). A clear difference, in terms of a decrease equal to 24% on average, was observed at 2% and 10% barley inclusions as well as at 10% bran inclusion as compared with semolina bread. Considering the other percentage of inclusions, an average decrease in specific volumes of 15.5% and 12.6% for barley and bran, respectively, compared with semolina bread, was evidenced. The results of this study were in line with other authors [41,42,43], who observed slight decreases or similar values to the control sample of specific volume in wheat breads incorporating barley/soybean flours or chia seeds. The differences observed among samples could be due to the introduction of fiber-rich products into the dough that negatively affects the formation of gluten, reducing its ability to retain gases [44,45,46]. Instead, others [40,47] found higher bread volume due to the high MW β-glucans found in barley/oat flours or added as β-glucan isolate, which may, in turn, stabilize gas cells by increasing the viscosity of the doughs.

The same behavior observed for specific volumes was also found for height and specific weight among the control and the other bread samples.

No significant differences in crumb porosity were observed among the bread samples compared with semolina bread, except for the bread containing 2% thin bran supplementation, in which the crumb porosity suffers deterioration, showing a non-homogeneous crumb. The results of this study disagree with [48,49], who observed increases in the porosity of the crumb, measured on the Dallmann scale, replacing wheat flour with brewer’s spent grain or fresh pumpkin pulp.

A decrease in hardness was observed in breads with barley/thin bran inclusion compared with bread control. Barley or thin bran addition to semolina decreased the hardness by 45.5%, on average at 5% and 7% barley inclusion, and by 39.4%, on average at 2% and 5% thin bran inclusion, compared with semolina bread. The results of this study agreed with Adamczyk et al. [41], who observed a reduction of hardness in bread by replacing 1 or 5% *w/w* whole chia seeds with wheat flour.

Finally, the moisture content ranged from 19.32–28.33% in barley supplementations, increasing with the increase of barley amount added up to reach similar values to bread control at 10% of barley inclusion (28.33% vs. 28.47%). In contrast, the inclusion of increasing thin bran levels determined a decrease in moisture, showing absolute values higher than semolina bread (30.35% vs. 28.47%, on average). Presumably, the different water hydration properties and profiles of the fiber blend polymers might justify the different behavior in water retention capacity and moisture of breads, reducing bread hardness [50,51].

Table 8 shows the evaluation of the physical properties of the bread samples produced using different types of supplementation: barley flour and thin bran. Two-factor ANOVA was not significant.

Table 9 shows the evaluation of the physical properties of the bread samples produced using different levels of supplementation: two-factor ANOVA (analysis of variance) referred to the different percentages of integration of two flours. The specific volume and height of the loaves decrease as the percentage of integration increases. The specific weight and moisture parameters show the opposite trend. The hardness remained almost unchanged, while the porosity was not significant.

### 2.3. Results of β-Glucans of Breads with Different Percent of Barley/Thin Bran Flour Compared with Control Bread

Concerning the β-glucans content, after baking, supplemented bread showed an increase in β-glucans compared with semolina bread (Table 10), independently from the enrichment level. In fact, the bread obtained with the inclusion of barley or thin bran showed twofold β-glucan increases at 2% barley supplementation up to threefold at 10% barley, while 1.7-fold and 2.7-fold increases were recorded at 7% and 10% thin bran supplementation. Indeed, it is well documented that many processing methods, such as milling, germination, cooking, baking, extrusion roasting, and freezing, can affect the stability, solubility, and viscosity of β-glucan differently [52]. Johansson et al. [53] observed decreases of β-glucan after baking, whilst Cavallero et al. [54] and Blandino et al. [55] reported that the bread-baking process did not reduce the β-glucan content. The increased values reported in this study, as observed by Izydorczyk et al. [56], could be due to hydrothermal treatment (steaming) during baking that, although not affecting the extractability of β-glucans, prevent their enzymatic hydrolysis, with no change or disrupt the β-glucan to form other aggregates. The same result was observed by other authors [57] in pasta obtained by replacing semolina with barley flour rich in β-glucan, in which cooking increased the extractability and the viscosity, determining its physiological effectiveness.

Table 11 shows the β-glucans contents (% *w/w*) of the enriched breads, based on the raw materials (thin bran and barley flour), as determined by the two-factor ANOVA. ANOVA was not significant.

Table 12 shows the β-glucans contents (% *w/w*) of the enriched breads, based on the raw materials (thin bran and barley flour) and on the level of inclusions, as determined by the two-factor ANOVA. As expected, as the percentage of integration increases, the content in β-glucans increases.

### 2.4. Color Indices in Crumb and Crust Breads Obtained with Different Formulations

Other important features of the bread samples are the crust and crumb color, which are also highly associated with bread consumers’ acceptance. Crumb color results (Table 13) reveal that the inclusion of barley or thin bran increased the brownness tone (100-L* values), the reddish tone (a* values), and the yellowness tone (b* values) in different ways. In contrast, crust color lighter breads (100-L* values) with a remarkable yellowness tone (b* values) and reddish tone (a* values) were obtained. The brownness tone in the crumb was highest at 10% barley inclusion, while the color at 2% barley level was similar to semolina bread; the brownness tone in the crust of bread with 10% barley inclusion was similar to semolina and lower in the other samples. The brownness, which is influenced by the flour type and extraction [58], was the result of the occurrence of a Maillard reaction during heat treatment and of the enzymatic oxidation—polyphenol oxidase and peroxidase—of phenolics to brown quinones [59]. The reddish tone in the crumb varied from less negative to strictly positive and/or higher values than the semolina bread ones, with a maximum obtained with 10% barley inclusion. Instead, the crust’s maximum value was reached at 2% bran inclusion. The yellowness tone increased differently at different flour inclusion, both in the crumb and crust.

Table 14 shows the evaluation of colorimetric parameters of the bread samples based on different types of supplementation, as determined by the two-factor ANOVA (analysis of variance). ANOVA was not significant.

Table 15 shows the evaluation of colorimetric parameters of the bread samples based on the level of inclusions, as determined by the two-factor ANOVA (analysis of variance). The three color parameters of the crumb increase as the percentage of integration increases.

Regarding the crust, the brown index follows the same trend, while the red and yellow indexes decrease as the percentage of integration increases.

## 3. Materials and Methods

### 3.1. Raw Materials

Durum wheat [*Triticum turgidum* L. subsp. *durum* (Desf.) Husn.] semolina and thin bran was kindly provided by ‘Valle del Dittaino’ Agricultural Cooperative Society a.r.l. (Enna, Italy), a local industrial bakery with a durum wheat mill (Golfetto, Padova, Italy). The barley flour used was bought from a local dealer (Somercom s.r.l., Viagrande, Catania, Italy).

The flour blends were made by substituting the 100% durum wheat semolina (ctrl) with whole barley flour or thin bran at 2%, 5%, 7%, and 10% (*w/w*).

### 3.2. Physico-Chemical Analyses of Raw Materials and Flour Blends

The β-glucan content of semolina and flour blends was determined enzymatically according to AACC 32.23.01 method [60] using the Megazyme β-glucan assay kit (Megazyme, Bray, Ireland) and was expressed as the percentage of flour weight on fresh weight (f.w.) basis.

Protein content was determined using the Kjeldahl method, according to the American Association of Cereal Chemists (AACC) approved method 46–13.01 [61]. The multiplication factors used were 5.7 for cereals.

Ash content was obtained following the ISO method 2171 [62].

The color parameters in the color space L*, a*, and b* were determined by Chroma meter CR-300 (Minolta, Osaka, Japan) under the illuminant D_65_. Brown index was calculated as 100-L*.

The analyses of the raw materials and flour blends were carried out in triplicate.

### 3.3. Technological Tests on Doughs of Semolina and Flour Blends

The Mixograph curves were obtained following the AACC method 54–40.02 [63] using a Mixograph National Mfg. Co. (Lincoln, NE, USA).

The Farinograph indices were determined according to the AACC 54–21 method [64] by a Farinograph (Brabender instrument, Duisburg, Germany) equipped with the software Farinograph^®^ (Brabender instrument, Duisburg, Germany). Water absorption needed to achieve the dough consistency of 500 ± 20 Brabender Units (B.U.) (A), dough development time (B), dough stability (CD), and consistency drop off after 12 min (E12) were measured according to the ICC methodology.

The measures were replicated three times.

### 3.4. Baking Test

The breadmaking test was performed on the semolina control and on flours obtained from each thesis according to the AACC 10–10.03 procedure (2000), as modified for durum wheat by Boggini and Pogna [65], into the baking time (18 min) and temperature (217 ± 4.24 °C), to obtain two loaves of about 140 g/each. Two independent replicate baking experiments were carried out.

Hence, a total of 36 loaves were obtained, onto which the following traits were individually measured: volume, height, weight, crumb porosity, hardness, moisture, crumb, and crust color.

The specific volume and specific weight were calculated by comparing the loaf volume to its weight and the loaf weight to its volume. The loaf height was measured using a digital caliper (Digi-MaxTM, SciencewareR, NJ, USA). The crumb porosity was assessed according to the Dallmann scale [66].

The loaf hardness was measured using a texture analyzer (Zwick Z 0.5 Roell, Ulm, Germany) equipped with an aluminum 8 mm diameter cylindrical probe.

The moisture content was determined by gravimetric analysis.

The CIE L*, a*, b* color parameters were measured for the crumbs in the transversely cut bread and on the crust surface, using a Chroma Meter (CR-200, Minolta) with illuminant D_65_. The measurements were replicated twice, except for the loaf hardness, which had in triplicate. The results of two loaves of a single batch for each thesis were averaged into one replicate value.

### 3.5. Statistical Analysis of Data

The statistical analysis was performed using the Statgraphics^®^ Centurion XVI software package (Statpoint Technologies, INC., The Plains, Virginia). One-factor and two-factor analysis of variance (ANOVA), followed by Tukey’s HSD test (*p* ≤ 0.05), was carried out on all physicochemical, technological, and breadmaking attributes. The two factors were considered: 1. the type of ingredient, 2. the amount of ingredient. A one-factor analysis determined the interaction of the factors studied, while a two-factor analysis analyzed each factor’s influence (or lack of influence) individually.

## 4. Conclusions

In general, although the Mixograph and Farinograph parameters were negatively affected by the inclusion of barley flour or thin bran and even exceeded the typical values of semolina control [56], the final bread quality showed a little reduction in specific volume, which is an important parameter for evaluating bread-making quality. Also, a decrease in hardness was observed, while other parameters, such as crumb porosity, remained unchanged.

After baking, an increase of β-glucans was observed in all samples, more evident at high barley flour/thin bran inclusion levels, showing that the heat treatment inactivated endogenous enzymes resulting in reduced β-glucans degradation.

Moreover, the variation of color tones, already significant at low levels of supplementation, was generally progressively more evident with the increase of flour added. As color is an important attribute that strongly influences consumer choice, high differences from bread without supplementation could be negatively considered. So, the inclusion of intermediate percentages of alternative cereals to semolina formulations could be a compromise to improve the nutritional properties of breads while maintaining the rheological performance and acceptability in terms of color similarity to semolina bread.

### Future Work

The study conducted so far has to be intended as a work to establish the technological conditions for optimal final product development. This is to identify the type of flour to add, different from re-milled semolina, giving the best results and the optimal percentage to be used. In the future, we will focus on bread’s nutritional, nutraceutical, sensory, and storage aspects, particularly the amount of extracted β-glucan and the glycemic responses to carbohydrate products.

## Figures and Tables

**Table 1:** Physicochemical features of raw materials and flour blends: one-factor ANOVA (analysis of variance) (data are means ± standard deviations).

Sample	β-Glucan Content (% *w/w*)	Moisture (% *w/w*)	Protein Content (% *w/w*)	Ash (% *w/w*)	Brown Index (100-L*)	Red Index (a*)	Yellow Index (b*)
*Pure flours*							
100% semolina (ctrl)	0.31 ± 0.03 d	11.80 ± 0.10 def	14.48 ± 0.06 ab	0.64 ± 0.09 c	10.21 ± 0.08 f	−2.39 ± 0.02 fg	17.20 ± 0.05 bc
Barley flour	10.61 ± 0.41 a	10.10 ± 0.20 g	8.88 ± 0.19 e	1.92 ± 0.07 b	12.14 ± 0.03 c	−0.47 ± 0.03 b	12.30 ± 0.02 f
Thin bran	1.19 ± 0.16 b	14.73 ± 0.06 a	14.87 ± 0.29 ab	2.70 ± 0.29 a	28.28 ± 0.05 a	3.68 ± 0.10 a	21.81 ± 0.03 a
*Blends*							
10% barley flour	1.58 ± 0.06 b	11.00 ± 0.26 f	12.84 ± 0.09 d	0.80 ± 0.08 c	11.05 ± 0.04 def	−1.62 ± 0.04 cde	16.03 ± 0.55 e
7% barley flour	1.45 ± 0.07 b	11.17 ± 0.25 ef	13.24 ± 0.15 cd	0.72 ± 0.10 c	10.58 ± 0.06 ef	−1.94 ± 0.05 def	16.34 ± 0.10 de
5% barley flour	1.06 ± 0.02 bc	11.20 ± 0.10 ef	13.50 ± 0.45 cd	0.68 ± 0.09 c	10.33 ± 0.04 ef	−2.06 ± 0.04 defg	16.38 ± 0.04 cde
2% barley flour	0.53 ± 0.02 cd	11.40 ± 0.10 def	14.00 ± 0.12 bc	0.66 ± 0.08 c	10.77 ± 0.07 f	−2.17 ± 0.03 efg	17.40 ± 0.02 b
10% thin bran	0.46 ± 0.02 d	12.83 ± 0.31 b	14.99 ± 0.10 a	0.84 ± 0.07 c	13.25 ± 0.78 b	−1.35 ± 0.09 c	16.15 ± 0.13 de
7% thin bran	0.40 ± 0.05 d	12.70 ± 0.20 bc	14.77 ± 0.11 ab	0.82 ± 0.04 c	12.10 ± 0.05 cd	−1.51 ± 0.04 cd	16.70 ± 0.04 bcde
5% thin bran	0.38 ± 0.04 d	12.25 ± 0.25 bcd	14.62 ± 0.16 ab	0.75 ± 0.06 c	11.60 ± 0.02 cde	−1.76 ± 0.02 cde	16.80 ± 0.05 bcde
2% thin bran	0.29 ± 0.04 d	11.90 ± 0.10 cde	14.43 ± 0.17 ab	0.71 ± 0.03 c	11.38 ± 0.06 cde	−2.54 ± 0.39 g	16.97 ± 0.23 bcd

Values followed by the same small letter within the same column are not significantly different (*p* < 0.01).

**Table 2:** Physicochemical features of the two flours: thin bran and barley flour: two-factor ANOVA (analysis of variance) (data are means ± standard deviations).

Sample	β-Glucan Content (% *w/w*)	Moisture (% *w/w*)	Protein Content (% *w/w*)	Ash (% *w/w*)	Brown Index (100-L*)	Red Index (a*)	Yellow Index (b*)
Thin bran	0.54 ± 0.35	12.88 ± 1.03	14.74 ± 0.25	1.16 ± 0.80	15.32 ± 6.75	−0.70 ± 2.31	17.69 ± 2.15
Barley flour	3.05 ± 3.94	10.97 ± 0.50	12.49 ± 1.92	0.96 ± 0.51	10.97 ± 0.65	−1.65 ± 0.64	15.69 ± 1.83

**Table 3:** Physicochemical features to the different percentages of integration of two flours: two-factor ANOVA (analysis of variance) (data are means ± standard deviations).

Integration Percentage	β-Glucan Content (% *w/w*)	Moisture (% *w/w*)	Protein Content (% *w/w*)	Ash (% *w/w*)	Brown Index (100-L*)	Red Index (a*)	Yellow Index (b*)
100%	5.90 ± 5.17 a	12.41 ± 2.54 a	11.88 ± 3.29 b	2.31 ± 0.46 a	20.21 ± 8.84 a	1.61 ± 2.27 a	17.05 ± 5.21 ab
2%	0.41 ± 0.13 c	11.65 ± 0.29 b	14.21 ± 0.27 a	0,68 ± 0.06 b	11.08 ± 0.34 c	−2.35 ± 0.32 d	17.19 ± 0.27 a
5%	0.72 ± 0.38 bc	11.73 ± 0.60 b	14.06 ± 0.68 a	0,71 ± 0.07 b	10.96 ± 0.70 c	−1.91 ± 0.17 c	16.59 ± 0.23 bc
7%	0.92 ± 0.58 b	11.93 ± 0.86 ab	14.01 ± 0.85 a	0,77 ± 0.09 b	11.34 ± 0.83 c	−1.73 ± 0.24 bc	16.52 ± 0.21 bc
10%	1.02 ± 0.62 b	11.92 ± 1.04 ab	13.91 ± 1.18 a	0,82 ± 0.06 b	12.15 ± 1.30 b	−1.49 ± 0.16 b	16.09 ± 0.36 c

Values followed by the same small letter within the same column are not significantly different (*p* < 0.01).

**Table 4:** Main technological parameters on semolina and flour inclusions: one-factor ANOVA (analysis of variance) (data are means ± standard deviations).

Mixograph			Farinograph			
Sample	Mixing Time (min)	Peak Dough Height (M.U.) *	Farinograph Absorption at 500 B.U. (%) **	Development Time (min)	Dough Stability (min)	Softening Index (B.U.)
100% semolina (ctrl)	2.68 ± 0.06 e	6.37 ± 0.04 a	60.59 ± 0.09 h	1.79 ± 0.08 d	3.29 ± 0,08 g	53.00 ± 2.00 a
10% barley flour	5.13 ± 0.11 a	5.18 ± 0.07 c	73.23 ± 0.06 a	2.15 ± 0.13 c	6.73 ± 0.06 d	52.67 ± 2.52 ab
7% barley flour	4.34 ± 0.04 b	5.53 ± 0.11 b	68.59 ± 0.09 b	4.55 ± 0.09 a	8.78 ± 0.08 c	42.67 ± 1.15 cb
5% barley flour	4.11 ± 0.05 b	5.78 ± 0.08 b	67.13 ± 0.06 c	4.08 ± 0.03 b	10.08 ± 0.08 b	35.33 ± 0.58 c
2% barley flour	3.50 ± 0.06 c	6.42 ± 0.03 a	63.05 ± 0.18 g	1.53 ± 0.06 de	5.22 ± 0.10 e	56.67 ± 1.53 a
10% thin bran	3.12 ± 0.04 d	6.18 ± 0.07 a	65.32 ± 0.03 d	1.67 ± 0.06 de	18.75 ± 0.05 a	14.33 ± 0.58 d
7% thin bran	2.40 ± 0.06 f	5.73 ± 0.12 b	64.88 ± 0.07 e	1.62 ± 0.03 de	18.73 ± 0.15 a	14.33 ± 1.53 d
5% thin bran	2.22 ± 0.04 f	5.78 ± 0.08 b	64.53 ± 0.06 e	1.78 ± 0.03 d	18.72 ± 0.07 a	22.67 ± 0.58 d
2% thin bran	3.17 ± 0.04 d	5.52 ± 0.03 b	63.68 ± 0.07 f	1.43 ± 0.06 e	3.78 ± 0.07 f	56.67 ± 1.15 a

* Mixograph units; ** Brabender units. Values followed by the same small letter within the same column are not significantly different (*p* < 0.01).

**Table 5:** Main technological parameters: two-factor ANOVA (analysis of variance) referred to the two flours: thin bran and barley flour (data are means ± standard deviations).

Mixograph			Farinograph		
Sample	Mixing Time (min)	Peak Dough Height (M.U.) *	Farinograph Absorption at 500 B.U. (%) **	Development Time (min)	Dough Stability (min)
Thin bran	2.73 ± 0.44	5.81 ± 0.26	64.60 ± 0.63	1.63 ± 0.14	15.00 ± 6.77
Barley flour	4.27 ± 0.61	5.73 ± 0.48	68.00 ± 3.80	3.08 ± 1.33	7.70 ± 1.95

* Mixograph units; ** Brabender units.

**Table 6:** Main technological parameters: two-factor ANOVA (analysis of variance) referred to the different percentages of integration of two flours (data are means ± standard deviations).

Mixograph			Farinograph		
Integration Percentage	Mixing Time (min)	Peak Dough Height (M.U.) *	Farinograph Absorption at 500 B.U. (%) **	Development Time (min)	Dough Stability (min)
2%	3.33 ± 0.19 b	5.97 ± 0.49 a	63.37 ± 0.37 d	1.48 ± 0.08 c	4.50 ± 0.79 d
5%	3.17 ± 1.03 c	5.78 ± 0.07 ab	65.83 ± 1.43 c	2.93 ± 1.26 a	14.40 ± 4.73 a
7%	3.37 ± 1.07 b	5.63 ± 0.15 b	66.73 ± 2.04 b	3.08 ± 1.61 a	13.76 ± 5.45 b
10%	4.12 ± 1.11 a	5.68 ± 0.55 b	69.27 ± 4.33 a	1.91 ± 0.28 b	12.74 ± 6.59 c

* Mixograph units; ** Brabender units. Values followed by the same small letter within the same column are not significantly different (*p* < 0.01).

**Table 7:** Evaluation of physical properties and moisture of the bread samples produced using different types and levels of supplementation: one-factor ANOVA (analysis of variance) (data are means ± standard deviations).

Sample	Specific Volume (cm^3^/g)	Height (mm)	Specific Weight (g/cm^3^)	Porosity (1–8)	Hardness (N)	Moisture (%)
100% semolina (ctrl)	2.9 ± 0.07 a	76.0 ± 0.00 a	0.4 ± 0.01 c	7 ± 0.3 a	16.85 ± 0.27 a	28.47 ± 0.01 e
10% barley flour	2.1 ± 0.03 c	64.4 ± 1.63 b	0.5 ± 0.01 a	7 ± 0.4 a	13.81 ± 1.42 ab	28.33 ± 0.02 e
7% barley flour	2.4 ± 0.03 abc	70.2 ± 0.14 ab	0.4 ± 0.01 abc	6 ± 0.3 ab	10.43 ± 1.86 b	22.93 ± 0.01 f
5% barley flour	2.5 ± 0.05 abc	70.4 ± 0.00 ab	0.4 ± 0.01 abc	5 ± 0.4 ab	7.94 ± 1.64 b	20.13 ± 0.02 g
2% barley flour	2.3 ± 0.10 bc	66.6 ± 3.04 b	0.4 ± 0.02 ab	6 ± 0.3 ab	11.08 ± 0.32 ab	19.32 ± 0.03 h
10% thin bran	2.2 ± 0.08 bc	65.1 ± 1.84 b	0.5 ± 0.02 ab	6 ± 0.4 ab	11.85 ± 2.46 ab	28.74 ± 0.02 d
7% thin bran	2.4 ± 0.02 abc	67.1 ± 1.20 ab	0.4 ± 0.00 abc	6 ± 0.4 ab	11.33 ± 0.58 ab	30.61 ± 0.01 b
5% thin bran	2.5 ± 0.10 abc	68.9 ± 3.11 ab	0.4 ± 0.02 abc	5 ± 0.4 ab	10.55 ± 1.18 b	30.32 ± 0.03 c
2% thin bran	2.7 ± 0.05 ab	72.7 ± 1.06 ab	0.4 ± 0.01 bc	4 ± 0.71 b	9.85 ± 0.27 b	31.74 ± 0.02 a

Values followed by the same small letter within the same column are not significantly different (*p* < 0.01).

**Table 8:** Evaluation of physical properties of the bread samples produced using different types of supplementation: two-factor ANOVA (analysis of variance) referred to the two flours: barley flour and thin bran (data are means ± standard deviations).

Sample	Specific Volume (cm^3^/g)	Height (mm)	Specific Weight (g/cm^3^)	Porosity (1–8)	Hardness (N)	Moisture (%)
Barley flour	2.32 ± 0.18	67.88 ± 3.02	0.43 ± 0.03	6.25 ± 1.04	10.90 ± 2.50	22.68 ± 3.77
Thin bran	2.44 ± 0.17	68.43 ± 3.33	0.41 ± 0.03	6.50 ± 1.31	10.82 ± 1.49	30.35 ± 1.15

**Table 9:** Evaluation of physical properties of the bread samples produced using different levels of supplementation: two-factor ANOVA (analysis of variance) referred to the different percentages of integration of two flours (data are means ± standard deviations).

Sample	Specific Volume (cm^3^/g)	Height (mm)	Specific Weight (g/cm^3^)	Porosity (1–8)	Hardness (N)	Moisture (%)
2%	2.46 ± 0.24 ab	69.60 ± 3.98 a	0.41 ± 0.04 ab	6.00 ± 2.83	10.46 ± 2.09 ab	25.53 ± 7.17 c
5%	2.50 ± 0.08 a	69.65 ± 1.99 a	0.40 ± 0.01 b	6.50 ± 2.12	9.25 ± 1.33 b	25.22 ± 5.89 c
7%	2.40 ± 0.03 ab	68.63 ± 1.95 ab	0.42 ± 0.01 ab	6.50 ± 0.71	10.88 ± 1.92 ab	26.77 ± 4.43 b
10%	2.17 ± 0.08 b	64.73 ± 1.48 b	0.46 ± 0.02 a	6.50 ± 0.71	12.83 ± 2.09 a	28.53 ± 0.24 a

Values followed by the same small letter within the same column are not significantly different (*p* < 0.01).

**Table 10:** β-glucans content of the semolina bread with different bread sample formulations (data are means ± standard deviations).

Sample	β-Glucans (% *w/w*)
100% semolina (ctrl)	0.61 ± 0.04 d
10% barley flour	1.85 ± 0.10 a
7% barley flour	1.59 ± 0.11 ab
5% barley flour	1.58 ± 0.02 ab
2% barley flour	1.27 ± 0.09 bc
10% thin bran	1.65 ± 0.08 a
7% thin bran	1.03 ± 0.08 c
5% thin bran	0.65 ± 0.03 d
2% thin bran	0.62 ± 0.02 d

Values followed by the same small letter within the same column are not significantly different (*p* < 0.01).

**Table 11:** β-Glucans contents (% *w/w*) of the enriched breads, based on the raw materials (thin bran and barley flour), as determined by the two-factor ANOVA (analysis of variance).

Sample	β-Glucans (% *w/w*)
Thin bran flour	0.99 ± 0.43
Barley flour	1.57 ± 0.23

**Table 12:** β-Glucans contents (% *w/w*) of the enriched breads, based on the raw materials (thin bran and barley flour) based on the level of inclusions, as determined by the two-factor ANOVA (analysis of variance).

Integration Percentage	β-Glucans (% *w/w*)
2%	0.95 ± 0.36 c
5%	1.11 ± 0.51 bc
7%	1.31 ± 0.32 b
10%	1.75 ± 0.14 a

Values followed by the same small letter within the same column are not significantly different (*p* < 0.01).

**Table 13:** Colorimetric parameters on different bread formulations: one-factor ANOVA (analysis of variance) (data are means ± standard deviations).

Sample	Crumb			Crust		
	Brown Index (100-L)	Red Index (a*)	Yellow Index (b*)	Brown Index (100-L)	Red Index (a*)	Yellow Index (b*)
100% semolina (ctrl)	25.80 ± 0.87 d	−3.02 ± 0.16 e	21.66 ±0.50 de	64.03 ± 0.08 a	12.38 ± 0.20 d	17.04 ± 0.05 e
10% barley flour	33.85 ± 0.17 a	1.50 ± 0.17 a	22.85 ± 0.23 bcde	58.84 ± 1.18 b	12.68 ± 0.32 cd	21.56 ± 1.28 d
7% barley flour	30.92 ± 1.14 ab	0.42 ± 0.05 b	22.38 ± 0.23 cde	56.77 ± 1.39 bc	14.90 ± 0.43 ab	25.73 ± 2.15 bcd
5% barley flour	30.58 ± 2.08 abc	−0.44 ± 0.04 c	21.04 ± 0.83 e	49.62 ± 0.50 d	14.57 ± 0.17 ab	30.32 ± 0.35 a
2% barley flour	26.19 ± 1.13 cd	−1.75 ± 0.06 e	23.74 ± 0.57 abcd	51.32 ± 1.99 d	15.40 ± 0.77 ab	28.62 ± 0.25 abc
10% thin bran	29.10 ± 0.20 bcd	−0.45 ± 0.04 c	25.50 ± 0.07 a	52.93 ± 0.68 cd	14.36 ± 0.06 bc	25.14 ± 0.12 cd
7% thin bran	27.29 ± 0.69 bcd	−1.27 ± 0.02 d	24.35 ± 0.27 abc	50.41 ± 0.33 d	14.52 ± 0.61 b	28.56 ± 0.36 abc
5% thin bran	29.09 ±0.71 bcd	−1.61 ± 0.30 d	24.83 ± 0.45 ab	50.69 ± 1.20 d	15.44 ± 0.08 ab	29.57 ± 1.28 ab
2% thin bran	27.14 ± 0.31 bcd	−2.47 ± 0.14 e	22.59 ± 0.90 bcde	51.66 ± 1.16 d	16.24 ± 0.09 a	30.84 ± 0.76 a

Values followed by the same small letter within the same column are not significantly different (*p* < 0.01).

**Table 14:** Evaluation of colorimetric parameters of the bread samples based on different types of supplementation, as determined by the two-factor ANOVA (analysis of variance) (data are means ± standard deviations).

Sample	Crumb			Crust		
	Brown Index (100-L)	Red Index (a*)	Yellow Index (b*)	Brown Index (100-L)	Red Index (a*)	Yellow Index (b*)
Thin bran	30.38 ± 1.08	−0.07 ± 0.77	22.50 ± 1.21	54.14 ± 1.29	14.39 ± 0.83	26.56 ± 2.30
Barley flour	28.15 ± 3.07	−1.45 ± 1.25	24.32 ± 1.11	51.42 ± 4.13	15.14 ± 1.15	28.53 ± 3.63

**Table 15:** Evaluation of colorimetric parameters of the bread samples based on the level of inclusions, as determined by the two-factor ANOVA (analysis of variance) (data are means ± standard deviations).

Integration Percentage	Crumb			Crust		
	Brown Index (100-L)	Red Index (a*)	Yellow Index (b*)	Brown Index (100-L)	Red Index (a*)	Yellow Index (b*)
2%	26.67 ± 0.90 b	−2.11 ± 0.40 d	23.16 ± 0.92 ab	51.49 ± 1.47 bc	15.82 ± 0.67 a	29.73 ± 1.31 a
5%	29.83 ± 1.61 a	−1.03 ± 0.67 c	22.93 ± 2.16 b	50.15 ± 1.01 c	15.01 ± 0.49 a	29.95 ± 0.93 a
7%	29.10 ± 2.16 ab	−0.43 ± 0.93 b	23.36 ± 1.10 ab	53.59 ± 3.60 ab	14.71 ± 0.52 a	27.15 ± 2.07 a
10%	31.48 ± 2.60 a	0.53 ± 1.07 a	24.18 ± 1.46 a	55.89 ± 3.35 a	13.52 ± 0.94 b	23.35 ± 2.12 b

Values followed by the same small letter within the same column are not significantly different (*p* < 0.01).

## Data Availability

Not applicable.

## References

[1] Sissons M.J. (2008). Role of durum wheat composition on the quality of pasta and bread. Foods.

[2] Ferdousi R., Rouhi M., Mohammadi R., Mortazavian A.M., Khosravi-Darani K., Rad A.H. (2013). Evaluation of probiotic survivability in yogurt exposed to cold chain interruption. Iran J. Pharm. Res..

[3] Zhu F., Du B., Bian Z., Xu B. (2015). Beta-glucans from edible and medicinal mushrooms: Characteristics, physicochemical and biological activities. J. Food Compos. Anal..

[4] Tessari P., Lante A. (2017). A multifunctional bread rich in beta glucans and low in starch improves metabolic control in type 2 diabetes: A controlled trial. Nutrients.

[5] Erkan H., Çelik S., Bilgi B., Köksel H. (2006). A new approach for the utilization of barley in food products: Barley tarhana. Food Chem..

[6] Gill S., Vasanthan T., Ooraikul B., Rossnagel B. (2002). Wheat bread quality as influenced by the substitution of waxy and regular barley flour in their native and extruded forms. J. Cereal. Sci..

[7] Finocchiaro F., Ferrari B., Gianinetti A., Scazzina F., Pellegrini N., Caramanico R., Salati C., Shirvanian V., Stanca A.M. (2012). Effects of barley b-glucan enriched flour fractions on the glycaemic index of bread. Int. J. Food Sci. Nutr..

[8] Lazaridou A., Biliaderis C.G. (2007). Molecular aspects of cereal b-glucan functionality: Physical properties, technological applications and physiological effects. J. Cereal. Sci..

[9] Ereifej K.I., Al-Mahasneh M.A., Rababah T.M. (2006). Effect of barley flour on quality of balady bread. Int. J. Food Pro..

[10] Alu’datt M.H., Rababah T., Al-Rabadi G.J., Ereifej K., Gammoh S., Masadeh N., Torley P.J. (2014). Effects of barley flour and barley protein isolate addition on rheological and sensory properties of pita bread. J. Food Qual..

[11] Tosh S.M., Brummer Y., Miller S.S., Regand A., Defelice C., Duss R., Wolever T.M.S., Wood P.J. (2010). Processing affects the physicochemical properties of b-glucan in oat bran cereal. J. Agric. Food Chem..

[12] Johansson C.-G., Siljeström M., Asp N.-G. (1984). Dietary fibre in bread and corresponding flours-formation of resistant starch during baking. Z. Lebensm. Unters. Forsch..

[13] Djurle S., Andersson A.A.M., Andersson R. (2018). Effects of baking on dietary fibre, with emphasis on b-glucan and resistant starch, in barley breads. J. Cereal. Sci..

[14] Campbell G.M., Ross M., Motoi L. (2008). Bran in Bread: Effects of Particle Size and Level of Wheat and Oat Bran on Mixing, Proving and Baking.

[15] Alzuwaid N.T., Pleming D., Fellows C.M., Laddomada B., Sissons M. (2021). Influence of Durum Wheat Bran Particle Size on Phytochemical Content and on Leavened Bread Baking Quality. Foods.

[16] Sibakov J., Lehtinen P., Poutanen K., Delcour J.A., Poutanen K. (2013). 8—Cereal brans as dietary fibre ingredients. Woodhead Publishing Series in Food Science, Technology and Nutrition, Fibre-Rich and Wholegrain Foods.

[17] Tricase C., Amicarelli V., Lamonaca E., Leonardo Rana R., Tadele Z. (2018). Economic analysis of the barley market and related uses. Grasses as Food and Feed.

[18] Poutanen K., Katina K., Heiniö R.-L., Zhou W., Hui Y.H., De Leyn I., Pagani M.A., Rosell C.M., Selman J.D., Therdthai N. (2014). Bakery Products Science and Technology.

[19] El-Taib H.I., Rizk I.R.S.A., Yousif E.I., Hassan A.A. (2018). Effect of barley flour on wheat bread quality. Int. J. Food Prop..

[20] Packkia-Doss P.P., Chevallier S., Pare A., Le-Bail A. (2019). Effect of supplementation of wheat bran on dough aeration and final bread volume. J. Food Eng..

[21] Manach C., Scalbert A., Morand C., Rémésy C., Jiménez L. (2004). Polyphenols: Food sources and bioavailability. Am. J. Clin. Nutr..

[22] Holtekjølen A.K., Knutsen S.H. (2011). Antioxidant activity and phenolics in breads with added barley flour. Flour and Breads and their Fortification in Health and Disease Prevention.

[23] Andersson A.A., Armö E., Grangeon E., Fredriksson H., Andersson R., Åman P. (2004). Molecular weight and structure units of (1→ 3, 1→ 4)-β-glucans in dough and bread made from hull-less barley milling fractions. J. Cereal. Sci..

[24] Nishantha M.D.L.C., Zhao X., Jeewani D.C., Bian J., Nie X., Weining S. (2018). Direct comparison of β-glucan content in wild and cultivated barley. Int. J. Food Prop..

[25] Collar C., Angioloni A. (2014). Nutritional and functional performance of high β-glucan barley flours in breadmaking: Mixed breads versus wheat breads. Eur. Food Res. Technol..

[26] Messia M.C., Oriente M., Angelicola M., De Arcangelis E., Marconi E. (2019). Development of functional couscous enriched in barley β-glucans. J. Cereal. Sci..

[27] Pasqualone A., Laddomada B., Centomani I., Paradiso V.M., Minervini D., Caponio F., Summo C. (2017). Bread making aptitude of mixtures of re-milled semolina and selected durum wheat milling by-products. LWT.

[28] Basman A., Köksel H. (2001). Effects of barley flour and wheat bran supplementation on the properties and composition of Turkish flat bread, yufka. Eur. Food Res. Technol..

[29] Rani M., Singh G., Siddiqi Raashid A., Gill Balmeet S., Sogi Dalbir S., Bhat Mohd A. (2021). Comparative Quality Evaluation of Physicochemical, Technological, and Protein Profiling of Wheat, Rye, and Barley Cereals. Front Nutr..

[30] Mohebbi Z., Homayouni A., Azizi M.H., Hosseini S.J. (2018). Effects of beta-glucan and resistant starch on wheat dough and prebiotic bread properties. J. Food Sci. Technol..

[31] Astiz V., Guardianelli L.M., Salinas M.V., Brites C., Puppo M.C. (2023). High β-Glucans oats for healthy wheat breads: Physicochemical properties of dough and breads. Foods.

[32] Navrotskyi S., Guo G., Baenziger P.S., Xu L., Rose D.J. (2019). Impact of wheat bran physical properties and chemical composition on whole grain flour mixing and baking properties. J. Cereal. Sci..

[33] Clydesdale F.M. (1994). Optimizing the diet with whole grains. Crit. Rev. Food Sci. Nutr..

[34] Mehfooz T., Ali T.M., Arif S., Hasnain A. (2018). Effect of barley husk addition on rheological, textural, thermal and sensory characteristics of traditional flat bread (chapatti). J. Cereal. Sci..

[35] Tömösközi S., Békés F., Haraszi R., Gras P.W., Varga J., Salgó A. (2002). Application of Micro Z-arm dough mixer in wheat research—Effect of protein addition on mixing properties of wheat dough. Period. Polytech. Chem. Eng..

[36] Kaur L., Lukow O.M., Preston K.R., Malcolmson L.J. (2004). How well do early-generation quality tests predict flour performance?. Can. J. Plant Sci..

[37] Popa C.N., Tamba-Berehoius R.M., Culea R.E. (2015). The effect of added whole oat flour on some dough rheological parameters. Scientific Papers Series Management, Economic Engineering in Agriculture and Rural Development.

[38] Khatkar B.S., Bell A.E., Schofield J.D. (1996). Relationship between mixograph parameters and indices of wheat grain quality. J. Sci. Food Agric..

[39] Miś A., Grundas S., Dziki D., Laskowski J. (2012). Use of farinograph measurements for predicting extensograph traits of bread dough enriched with carob fiber and oat wholemeal. J. Food Eng..

[40] Skendi A., Biliaderis C., Papageorgiou M., Izydorczyk M. (2010). Effects of two barley b-glucan isolates on wheat flour dough and bread properties. Food Chem..

[41] Adamczyk G., Ivanišová E., Kaszuba J., Bobel I., Khvostenko K., Chmiel M., Falendysh N. (2021). Quality Assessment of Wheat Bread Incorporating Chia Seeds. Foods.

[42] Sullivan P., O’Flaherty J., Brunton N., Arendt E., Gallagher E. (2010). Fundamental rheological and textural properties of doughs and breads produced from milled pearled barley flour. Eur. Food Res. Technol..

[43] Dhingra S., Jood S. (2004). Effect of flour blending on functional, baking and organoleptic characteristics of bread. Int. J. Food Sci. Tech..

[44] Noort M.W., van Haaster D., Hemery Y., Schols H.A., Hamer R.J. (2010). The effect of particle size of wheat bran fractions on bread quality—Evidence for fibre-protein interactions. J. Cereal. Sci..

[45] Courtin C.M., Delcour J.A. (2002). Arabinoxylans and endoxylanases in wheat flour bread-making. J. Cereal Sci..

[46] Salmenkallio-Marttila M., Katina K., Autio K. (2001). Effects of bran fermentation on quality and microstructure of high-fiber wheat bread. Cereal Chem..

[47] Rieder A., Holtekjølen A.K., Sahlstrøm S., Moldestad A. (2012). Effect of barley and oat flour types and sourdoughs on dough rheology and bread quality of composite wheat bread. J. Cereal. Sci..

[48] Czubaszek A., Wojciechowicz-Budzisz A., Spychaj R., Kawa-Rygielska J. (2022). Effect of Added Brewer’s Spent Grain on the Baking Value of Flour and the Quality of Wheat Bread. Molecules.

[49] Różyło R., Gawlik-Dziki U., Dziki D., Jakubczyk A., Karaś M., Różyło K. (2014). Wheat bread with pumpkin (*Cucurbita maxima* L.) pulp as a functional food product. FTB.

[50] Collar C., Santos E., Rosell C.M. (2006). Significance of dietary fiber on the viscometric pattern of pasted and gelled flour-fiber blends. Cereal. Chem..

[51] Rosell C.M., Santos E., Collar C. (2009). Physico-chemical properties of dietary fibers from different sources: A comparative approach. Food Res. Int..

[52] Bobade H., Gupta A., Sharma S., Kour J., Nayik G.A. (2022). Chapter 20—Beta-glucan. Nutraceuticals and Health Care.

[53] Johansson L., Tuomainen P., Anttila H., Rita H., Virkki L. (2007). Effect of processing on the extractability of oat b-glucan. Food Chem..

[54] Cavallero A., Empilli S., Brighenti F., Stanca A.M. (2002). High (1 / 3, 1 / 4)-b-glucan barley fractions in bread making and their effects on human glycemic response. J. Cereal. Sci..

[55] Blandino M., Sovrani V., Marinaccio F., Reyneri A., Rolle L., Giacosa S., Locatelli M., Bordiga M., Travaglia F., Coïsson J.D. (2013). Nutritional and technological quality of bread enriched with an intermediated pearled wheat fraction. Food Chem..

[56] Izydorczyk M.S., Storsley J., Labossiere D., MacGregor A.W., Rossnagel B.G. (2000). Variation in total and soluble beta-glucan content in hulless barley: Effects of thermal, physical, and enzymic treatments. J. Agric. Food Chem..

[57] De Paula R., Abdel-Aal E.M., Messia M.C., Rabalski I., Marconi E. (2017). Effect of processing on the beta-glucan physicochemical properties in barley and semolina pasta. J. Cereal. Sci..

[58] Pourafshar S., Rosentrater K.A., Krishnan P.G. (2015). Using alternative flours as partial replacement of barbari bread formulation (traditional Iranian bread). J. Food Sci. Technol..

[59] Taranto F., Pasqualone A., Mangini G., Tripodi P., Miazzi M.M., Pavan S., Montemurro C. (2017). Polyphenol oxidases in crops: Biochemical, physiological and genetic aspects. Int. J. Molecular. Sci..

[60] American Association of Cereal Chemists (2000). Mixed-Linkage Beta-Glucan. Approved Methods of the American Association of Cereal. Chemists.

[61] American Association of Cereal Chemists (2000). Crude Protein—Micro-Kjeldahl Method. Approved Methods of the American Association of Cereal. Chemists.

[62] (2017). Cereals, Pulses and By-Products—Determination of ash yield by incineration.

[63] AACC International (2000). Approved Methods of Analysis, 11th Ed. Method 54–40.02. Mixograph Method.

[64] AACC (2000). Method AACC 54-21.01. Farinograph Method for Flour, Methods of the American Association of Cereal Chemists.

[65] Boggini G., Pogna N.E. (1989). The breadmaking quality and storage protein composition of Italian durum wheat. J. Cereal. Sci..

[66] https://portal.issn.org/resource/ISSN-L/2451-0769.

